# Mechanisms and Therapeutic Strategies of Macrophage Polarization in Intervertebral Disc Degeneration

**DOI:** 10.1002/jsp2.70065

**Published:** 2025-05-14

**Authors:** Kaiyuan Zheng, Siyu Wang, Meng Deng, Yaomin Luo, Wen Li, Lianlin Zeng, Yinxu Wang

**Affiliations:** ^1^ Department of Rehabilitation Medicine, Intensive Care Medicine Affiliated Hospital of North Sichuan Medical College Nanchong China; ^2^ Department of Clinical Laboratory The First People's Hospital of Guangyuan Guangyuan China; ^3^ Department of Rehabilitation Medicine Suining Central Hospital Suining China

**Keywords:** inflammation, intervertebral disc degeneration, macrophages polarization, mechanism, treatment

## Abstract

**Background:**

Intervertebral disc degeneration (IVDD) is a leading cause of low back pain (LBP), contributing significantly to global disability and productivity loss. Its pathogenesis involves complex processes, including inflammation, cellular senescence, angiogenesis, fibrosis, neural ingrowth, and sensitization. Emerging evidence highlights macrophages as central immune regulators infiltrating degenerated discs, with macrophage polarization implicated in IVDD progression. However, the mechanisms linking macrophage polarization to IVDD pathology remain poorly elucidated.

**Methods:**

A comprehensive literature review was conducted by searching major databases (PubMed, Web of Science, and Scopus) for studies published in the last decade (2014–2024). Keywords included “intervertebral disc degeneration,” “macrophage polarization,” “inflammation,” “senescence,” and “therapeutic strategies.” Relevant articles were selected, analyzed, and synthesized to evaluate the role of macrophage polarization in IVDD.

**Results:**

Macrophage polarization dynamically influences IVDD through multiple pathways. Pro‐inflammatory M1 macrophages exacerbate disc degeneration by amplifying inflammatory cytokines (e.g., TNF‐α, IL‐1β), promoting cellular senescence, and stimulating abnormal angiogenesis and neural ingrowth. In contrast, anti‐inflammatory M2 macrophages may mitigate degeneration by suppressing inflammation and enhancing tissue repair. Therapeutic strategies targeting macrophage polarization include pharmacological agents (e.g., cytokines, small‐molecule inhibitors), biologic therapies, gene editing, and physical interventions. Challenges persist, such as incomplete understanding of polarization triggers, lack of targeted delivery systems, and limited translational success in preclinical models.

**Conclusion:**

Macrophage polarization is a pivotal regulator of IVDD pathology, offering promising therapeutic targets. Future research should focus on elucidating polarization mechanisms, optimizing spatiotemporal control of macrophage phenotypes, and developing personalized therapies. Addressing these challenges may advance innovative strategies to halt or reverse IVDD progression, ultimately improving clinical outcomes for LBP patients.

## Graphical abstract

This article reviews the role of macrophage polarization in IVDD. It details how macrophage polarization influences various pathomechanisms of disc degeneration, including the inflammatory microenvironment, NPCs senescence, angiogenesis, fibrosis, and neural ingrowth and sensitization. Additionally, it provides an overview of current therapeutic strategies targeting macrophage polarization, identifies existing challenges, and outlines future research directions.

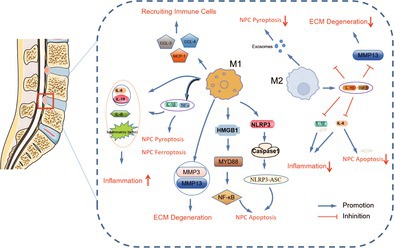

## 1 Introduction

More than 70% of people worldwide suffer from low back pain (LBP) to some degree, and it is a major contributor to both disability and lost productivity globally. Most people will at some point in their lives suffer from severe LBP, which has a significant socioeconomic impact [1]. One of the most prevalent causes of LBP is intervertebral disc degeneration (IVDD). Age‐related degeneration of the intervertebral discs is accelerated by smoking, obesity, mechanical stress, diabetes, and hereditary predisposition [2]. Furthermore, the incidence of IVDD is rising with the aging population and the increasing prevalence of sedentary lifestyles [3]. Degenerated discs exhibit structural changes, including decreased disc height, ruptured annulus fibrosus (AF), herniated nucleus pulposus (NP), reduced hydration, and diminished ability to absorb pressure loads [4]. These alterations lead to loss of disc function and may cause peripheral nerve compression and persistent inflammatory responses, resulting in chronic pain [5]. Current mainstream treatments for IVDD primarily aim to relieve symptoms and include surgical interventions, physical therapy, and pharmacological treatments. However, these approaches often fail to fundamentally improve disc degeneration or slow its progression [6]. Therefore, it is crucial to understand and target the mechanisms and etiology of IVDD.

Macrophages are immune cells that can polarize into various functional subtypes depending on their microenvironment [7]. The IVDD is a process of self‐repair after an inflammatory injury, and macrophages, as the predominant immune cells infiltrating the degenerated disc, are involved in this process [8]. Although the importance of macrophages in disc degeneration has been demonstrated, the specific mechanisms have not been systematically elucidated, and there is a lack of comprehensive summaries on how macrophage polarization affects disc degeneration. Therefore, this review elaborates on the macrophage polarization phenotype and delves into the mechanisms of macrophage polarization in IVDD. It also summarizes several therapeutic strategies targeting macrophage polarization, aiming to provide new insights and approaches for the treatment of IVDD.

## 2 Polarization Phenotype of Macrophages

As innate immune cells, macrophages are present in almost all tissues [9]. They perform diverse functions, including phagocytosis, regulation of inflammation, metabolism, development, and maintenance of tissue homeostasis [10, 11]. Macrophages, characterized by their plasticity and pluripotency, exhibit significant functional differences under varying microenvironments both in vivo and in vitro, enabling them to switch from one phenotype to another, a process known as macrophage polarization [12]. Based on their activation states and functional roles, macrophages are primarily classified into M1‐type (classically activated macrophages) and M2‐type (alternatively activated macrophages) (Table 1) [13].

**TABLE 1:** Polarization phenotypes of macrophages.

Polarization type	Activation	Molecular markers	Cytokines	Main functions
M1	LPS, IFN‐γ	CD86 CD197	IL‐1, IL‐6, IL‐12, IL‐23, TNF‐α	Phagocytosis, pro‐inflammatory, anti‐tumor, pathogen clearance
M2a	IL‐4, IL‐13	CD206 ARG1	IL‐10, TGF‐α	Anti‐inflammatory, tissue repair, angiogenesis, fibrosis‐promoting
M2b	LPS, IL‐1R ligands, TLR ligands	CD86	IL‐1, IL‐6, IL‐10, and TNF‐α	Anti‐inflammatory, immunomodulatory
M2c	IL‐10, TGF‐β, glucocorticoids	CD163	IL‐10, TGF‐β	Immunomodulatory, angiogenesis, Phagocytosis
M2d	Adenosine, TLR ligands	CD206, CD163, CD301, TIE2	IL‐10, VEGF	Anti‐inflammatory, angiogenesis

M1 macrophages are highly polarized in the presence of stimuli such as lipopolysaccharide (LPS) or interferon‐gamma (IFN‐γ). They secrete high levels of pro‐inflammatory cytokines, including IFN‐γ, IL‐1, IL‐6, IL‐12, IL‐23, and TNF‐α, and are involved in phagocytosis, pro‐inflammatory responses, anti‐tumor activity, and pathogen clearance. Their surface markers include CD197 (CCR7) and CD86 [14, 15, 16]. M2 macrophages can be induced by various activation signals, leading to different M2 phenotypes (M2a, M2b, M2c, M2d) [17]. M2a macrophages, activated by IL‐4 and IL‐13, secrete IL‐10 and TGF‐α, contributing to anti‐inflammation, tissue repair, angiogenesis, and fibrosis. Their surface markers include the mannose receptor MRc1 (CD206) and arginase‐1 (ARG1) [18]. M2b macrophages, activated by immune complexes, LPS, IL‐1R ligands, and TLR ligands, produce cytokines such as IL‐1, IL‐6, IL‐10, and TNF‐α, which have anti‐inflammatory and immunomodulatory effects, with CD86 as a surface marker [19]. M2c macrophages, activated by IL‐10, transforming growth factor‐beta (TGF‐β), and glucocorticoids, produce IL‐10 and TGF‐β, inhibiting inflammatory responses, participating in immunomodulation, promoting angiogenesis, and phagocytosis, with CD163 as a marker [20]. M2d macrophages are induced by adenosine and TLR ligands. Upon activation, they inhibit pro‐inflammatory cytokine production and induce anti‐inflammatory cytokines such as IL‐10 and vascular endothelial growth factor, exhibiting strong pro‐angiogenic capabilities, with markers including CD206, CD163, CD301, and TIE2 [21].

However, the traditional dichotomy of macrophages has been widely challenged in recent years [22]. It is now believed that macrophage polarization states are continuous rather than discrete, involving heterogeneous macrophage populations [23]. Studies have shown that human decidual macrophages in early pregnancy express both pro‐inflammatory and anti‐inflammatory cytokines [24]. Additionally, during wound repair, some macrophages display both M1 and M2 phenotypes, which do not conform to the traditional M1/M2 classification [10].

## 3 The Role of Macrophage Polarization in IVDD

**FIGURE 1:**
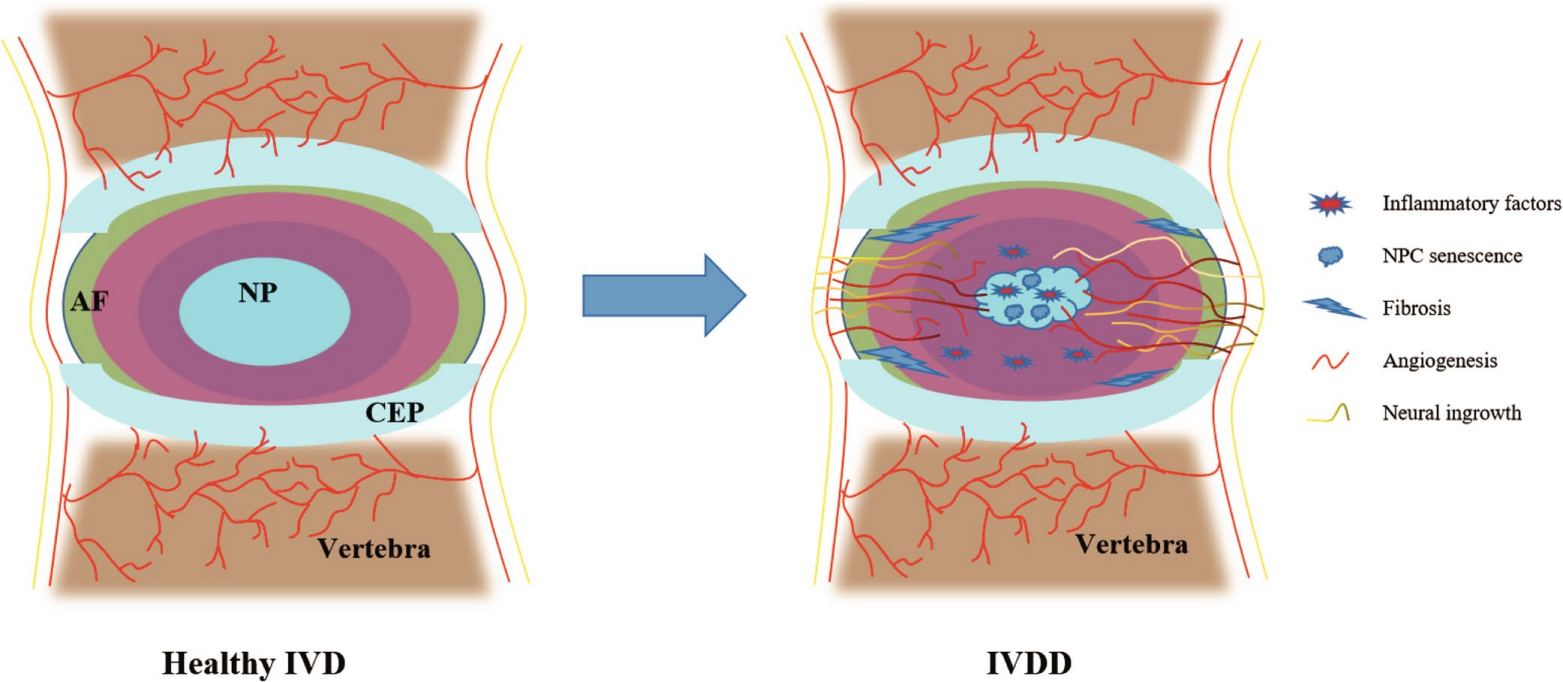
Pathological mechanisms of IVDD.

IVDD is a multifaceted process involving several pathological mechanisms, including inflammatory microenvironment‐induced Nucleus pulposus cells (NPCs) death, extracellular matrix (ECM) degradation, NP cell senescence, angiogenesis, fibrosis, and neural ingrowth and sensitization (Figure 1) [25, 26, 27]. Research indicates that the progression of disc degeneration is significantly influenced by the polarization states of macrophages [28]. Therefore, investigating the connection between macrophage polarization and these pathogenic processes is crucial for understanding IVDD.

### 3.1 Macrophage Polarization Regulates the Inflammatory Microenvironment

Inflammation is a critical driver of IVDD, contributing to NPCs death (including apoptosis, pyroptosis, and ferroptosis) and ECM degradation [29]. Understanding the link between macrophage polarization and inflammation is therefore essential for elucidating the pathomechanisms of disc degeneration.

M1 macrophages play a key role in regulating the inflammatory milieu. The inflammatory response is exacerbated by these macrophages' substantial secretion of pro‐inflammatory cytokines [28]. When Zhao et al. used LPS to promote M1 polarization in THP‐1 cells, they saw increased amounts of NLRP3 inflammasomes and pro‐inflammatory molecules such IL‐1β, IL‐6, TNF‐α, IL‐18, and High Mobility Group Box Protein 1 (HMGB1). M1 Macrophage‐derived exosomes use the LCN2/NF‐κB signaling axis to promote disc degeneration and increase NP cell senescence [30]. The conditioned medium from M1 macrophages facilitated apoptosis in myeloid cells via the HMGB1‐MyD88‐NF‐κB pathway and the NLRP3‐ASC‐Caspase1 inflammasome [31]. Additionally, the M1 macrophages' inflammatory factors trigger the intervertebral disc's synthesis of more pro‐inflammatory factors. TNF‐α, IL‐1β, and COX‐2 expression significantly increased in annulus fibrosus cells (AFC) and NPCs after Yang et al. treated them with conditioned media from IFN‐γ‐induced M1 macrophages [32]. IL‐1β and TNF‐α secreted by macrophages elevated IL‐8 and IL‐6 levels in AFC and NPCs, and upregulated inflammatory molecules such as inducible nitric oxide synthase, nitric oxide, prostaglandin E2, cyclooxygenase‐2, and TNF‐α, thereby exacerbating the inflammatory microenvironment [33, 34, 35]. Additionally, M1 macrophages produce chemokines that recruit more immune cells, further intensifying the inflammatory milieu. The interaction between M1 macrophages and the inflammatory microenvironment forms a cascade of mutual reinforcement. The inflammatory milieu recruits macrophages, promoting their M1 polarization and inducing their production of inflammatory factors [36, 37]. Tian's research demonstrated that degenerating myeloid cells trigger macrophage infiltration and M1 polarization through the CCL2/7‐CCR2 axis [38]. Similarly, Yang et al. found that conditioned media from LPS‐stimulated AFC and NPCs significantly elevated the levels of inflammatory factors TNF‐α, IL‐1β, and IL‐6, as well as chemokines CCL‐3, CCL‐4, and MCP‐1 in RAW 264.7 macrophages. This cascade effect contributed to the worsening of the intervertebral disc's inflammatory environment.

M1 macrophages also contribute to ECM degradation by modulating the inflammatory microenvironment. Matrix metalloproteinases (MMPs) are a large class of zinc‐containing and calcium‐dependent endopeptidases that play a crucial role in ECM degradation [39]. The conditioned medium of M1 macrophages, when co‐cultured with NPCs, promotes the expression of MMP‐3 and MMP‐13 in NPCs and AFC, and inhibits the cellular expression of type II collagen and aggregated proteoglycan, leading to ECM degradation [31, 32]. Additionally, M1 macrophages promote apoptosis, pyroptosis, and ferroptosis of NPCs by expanding the inflammatory microenvironment [31, 40, 41].

M2 macrophages also play a significant role in regulating the inflammatory microenvironment in disc degeneration, but unlike M1 macrophages, M2 macrophages exhibit anti‐inflammatory effects. M2 macrophages secrete a variety of anti‐inflammatory cytokines, such as IL‐10 and transforming growth factor‐β (TGF‐β) [42, 43]. These cytokines inhibit the inflammatory response through multiple pathways, including reducing the production of pro‐inflammatory cytokines [44, 45]. Additionally, M2 macrophages mitigate the degradation of the ECM caused by the inflammatory microenvironment. Conditioned media from M2 macrophages can suppress TNF‐α‐induced increases in IL‐1β, IL‐6, and MMP‐13 in myeloid cells while enhancing the expression of collagen II and the anabolic protein aggrecan [28, 46]. Liu et al. demonstrated that exosomes from M2c macrophages promote ECM synthesis under inflammatory conditions via the miR‐124/CILP/TGF‐β pathway, thereby attenuating disc degeneration [47].

Moreover, the anti‐inflammatory effects of M2 macrophages prevent myeloid cell death. In a TNF‐α‐induced myeloid degeneration model, conditioned medium from M2 macrophages reversed the expression of the inflammatory factor IL‐6 and inhibited NP apoptosis [46]. Similarly, exosomes from M2 macrophages inhibited NPCs pyroptosis via miR‐221‐3p [48]. Li's team investigated the effects of M2 macrophages on disc degeneration using Rheb‐deficient transgenic mice (RheBcKO mice with increased M2 macrophage expression) and in vitro co‐culture experiments. They found that M2 macrophages alleviated IVDD both in vivo and in vitro by inhibiting Rspo2 production, which reduced NPCs apoptosis under inflammatory conditions and inhibited ECM catabolism [49].

**FIGURE 2:**
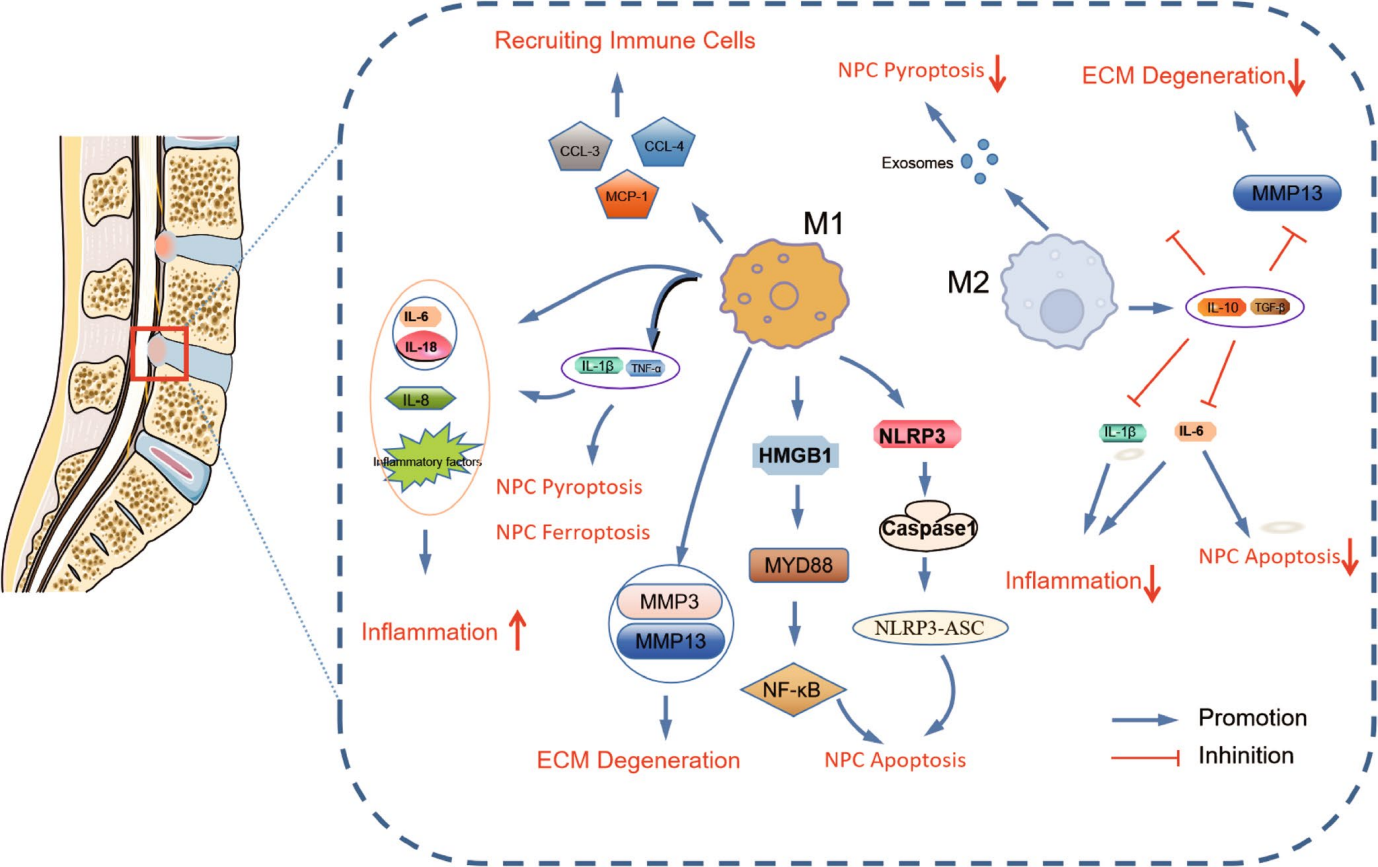
Diagram of the mechanism by which macrophage polarization influences IVDD by modulating the inflammatory microenvironment.

Inflammation is a major contributing factor to disc degeneration, and macrophage polarization influences this process by modulating the inflammatory microenvironment, affecting NPCs death, ECM metabolism, and thus disc degeneration (Figure 2).

### 3.2 Macrophage Polarization Modulates NPCs Senescence

Cellular senescence is an evolutionarily conserved biological process characterized by cell cycle arrest and the loss of replicative capacity. It can be categorized into replicative senescence, which results from telomere shortening and subsequent DNA damage response(DDR) after numerous cellular replications reaching the “Hayflick limit”, and stress‐induced senescence, which is triggered by various internal and external stressors such as radiation, oxidative stress, inflammation, ECM changes, and oncogenes, independent of the number of cell divisions [50, 51].

Macrophages and cellular senescence are closely related. Senescent cells release cytokines, such as chemokines and inflammatory substances, which attract macrophages to phagocytose and eliminate the accumulated senescent cells [52]. On the other hand, macrophages can influence cellular senescence themselves. Recent studies have shown that the senescent phenotype of macrophages can induce senescence in other cell types [50, 53, 54]. For instance, CX3CR1 macrophages attenuate adipose‐derived stem cell senescence by promoting the arginase 1‐eIF5A catalytic axis [55]. Senescent macrophages produce Grancalcin, which induces secondary senescence in stem/progenitor cells [56]. Cellular senescence is also influenced by macrophage polarization. M1 macrophages cause glomerular endothelial cell senescence in a diabetic kidney model via increasing intracellular reactive oxygen species (ROS) levels [57]. M2 macrophages induce hepatocyte senescence through the secretion of IL‐6 [58]. Furthermore, M2 macrophage‐derived TGF‐β induces senescence in adipose progenitor cells by causing DNA damage and increasing mitochondrial ROS, ultimately leading to age‐related lipodystrophy [59].

**FIGURE 3:**
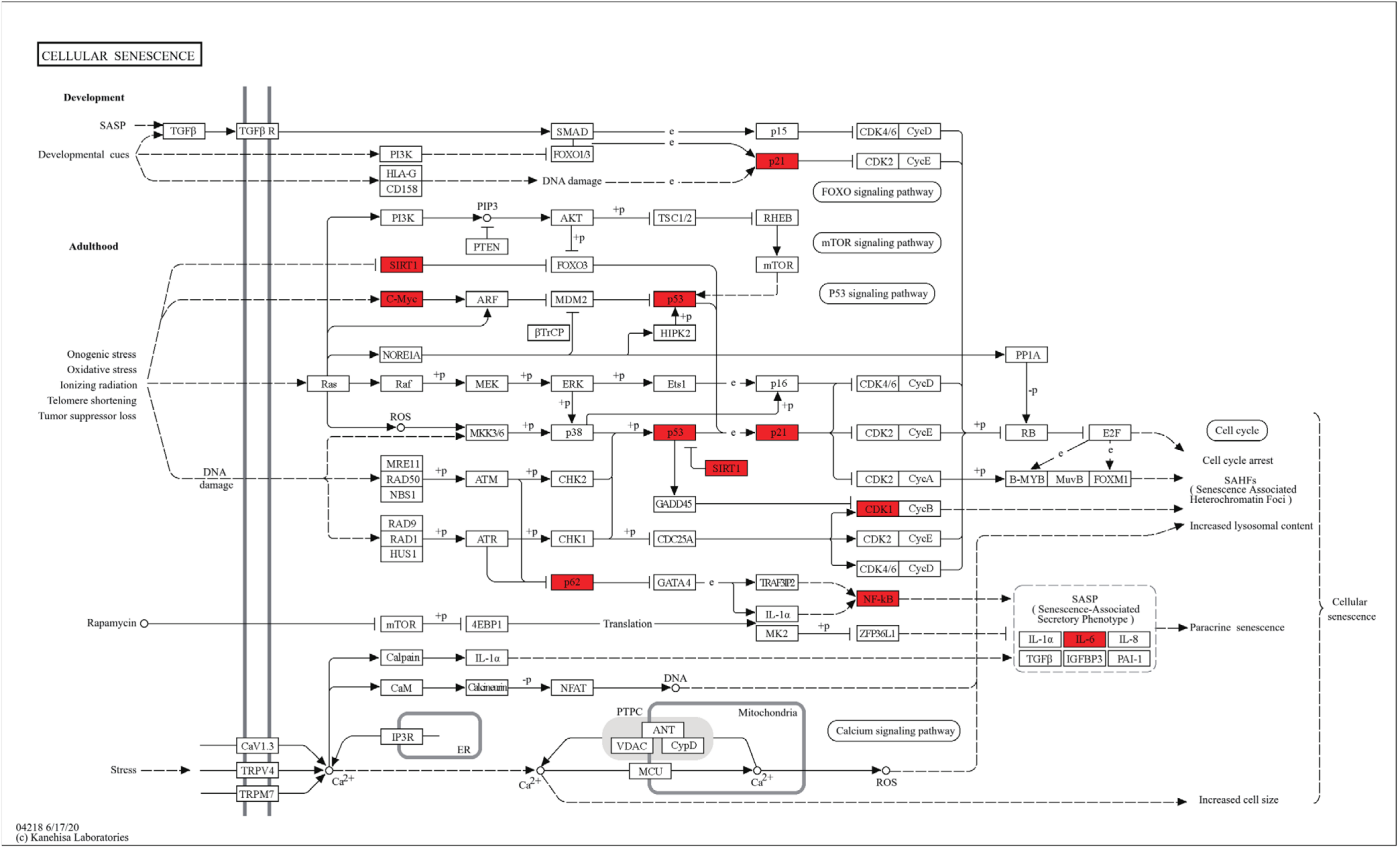
Regulation of molecular signaling pathways and related proteins in cellular senescence (this figure is from the KEGG database and has been described in the article acknowledgements).

In IVDD, NPCs senescence is a prominent feature, driven by inflammatory factors such as TNF‐α and IL‐6. Macrophage polarization plays a significant role in NP cell senescence (Figure 3). These cytokines are produced by invading M1 macrophages under degenerative circumstances, which exacerbate IVDD and NP cell senescence. By releasing lipocalin‐2 (LCN2), raising the frequency of senescence‐associated β‐galactosidase (SA‐β‐gal) positive cells, stopping the cell cycle, and activating P21 and P53, studies have demonstrated that exosomes from M1 macrophages can increase LPS‐induced NP cell senescence [30]. Increased ROS levels in degenerating discs further contribute to NP cell senescence. Evidence suggests that M1 macrophages produce ROS under hypoxic conditions, which may provide another mechanism through which M1 macrophages promote NP cell senescence. Furthermore, M1 macrophage‐induced NP cell senescence and myeloid cell senescence form a mutually reinforcing cascade. One study found that conditioned medium from M1 macrophages triggered NP cell senescence in a time‐dependent manner, while conditioned medium from senescent NP cells promoted macrophage migration and M1 polarization [60]. Breaking this cascade response may therefore be a key target for the treatment of disc degeneration.

Conversely, M2 macrophages exhibit an anti‐senescence phenotype in disc degeneration. Research indicates that conditioned media from M2 macrophages reduce the number of SA‐β‐gal positive cells and downregulate the expression of p16, p21, and p53 when co‐cultured with TNF‐α‐treated NP cells [46]. The role of M2 macrophages in NP cell senescence appears to contrast with their effects on cellular senescence in other tissues, likely due to the inflammatory microenvironment of senescent NP cells and the anti‐inflammatory nature of M2 macrophages. A small number of studies have found that M2 macrophage‐conditioned medium inhibits disc degeneration in an environment enriched with TNF‐α [46].

### 3.3 Macrophage Polarization Affects Angiogenesis

The largest avascular tissue in the body is the intervertebral disc. It is distinguished by the lack of blood vessels in the NP, with only a small number of blood vessels present in the upper and lower endplates and the outer third of the AF. In the NP, microvessel development is efficiently inhibited by an intact disc structure. Natural ECM components, such as aggregated proteoglycans (aggrecan) and high molecular weight hyaluronic acid (HA), inhibit endothelial cell adhesion and migration [61, 62]. Furthermore, healthy NP and AFC exert anti‐angiogenic effects by inducing endothelial cell apoptosis and inhibiting their migration [63]. During disc degeneration, this anti‐angiogenic mechanism is disrupted, leading to vascular invasion into the inner AF and even the NP. The extent of blood vessel invasion correlates positively with the degree of disc degeneration [64, 65]. The presence of blood vessels alters the hypoxic and immune‐protective environment of the intervertebral disc, creating conditions conducive to immune cell infiltration and further degeneration.

ECM modification, the activation of dormant endothelial cells (ECs), and the creation of novel vascular structures are all components of angiogenesis. Macrophages contribute significantly to this process in a number of ways. They release matrix MMPs, like MMP‐2 and MMP‐9, which break down and reorganize the extracellular matrix, promoting the growth of new blood vessels [66]. Additionally, macrophages release angiogenic factors that promote the maturation and stabilization of new blood vessels. For example, during liver tissue repair or regeneration, Kupffer cells (macrophages in the liver) stimulate angiogenesis to support tissue remodeling [67]. In cancer, tumor‐associated macrophages drive neovascularization, contributing to tumor growth [68, 69]. Similarly, during skin wound healing, macrophages are involved throughout the entire angiogenic process, including vascular sprouting, anastomosis, maturation, and reconstruction [70]. These examples underscore the pivotal regulatory role of macrophages in angiogenesis.

Interestingly, macrophages are not involved in the initial stages of angiogenesis during IVDD. Single‐cell transcriptome analysis of NP, AF, and cartilaginous endplate (CEP) cells from five healthy human intervertebral discs (Pfirrman class I) identified the presence of small blood vessels but no immune cells, indicating that vasculature appears prior to macrophage infiltration in degenerating discs [71]. Indeed, Early‐stage angiogenesis is primarily driven by disc cells themselves [72]. Degenerating intervertebral disc cells secrete various angiogenesis‐promoting factors, including VEGF, vascular cell adhesion molecule (VCAM), IL‐6, IL‐8, and several matrix MMPS, which act on endogenous endothelial cells to facilitate angiogenesis [73, 74, 75]. Thus, it is the disc cells, rather than macrophages, that primarily mediate early angiogenesis in disc degeneration.

Macrophages are recruited to the disc only after the initial formation of blood vessels, where they predominantly play a supporting and accumulating role in angiogenesis. M2‐type macrophages, in particular, are known to enhance angiogenesis by secreting pro‐angiogenic cytokines such as FGF2, CCL2, insulin‐like growth factor 1 (IGF1), and placental growth factor (PGF). These cytokines promote both the development and stabilization of new blood vessels. For example, M2 macrophages have been shown to induce pathological angiogenesis in intervertebral discs by downregulating cell migration‐inducing hyaluronidase 1 (CEMIP) [76].

Interestingly, only M2 macrophages have been documented to actively promote angiogenesis in degenerated intervertebral discs, whereas M1 macrophages have not been observed to have a similar effect. Although angiogenesis in degenerated discs involves various inflammatory factors, ROS, and nitric oxide (NO), which are associated with macrophages, there is no evidence to suggest that M1 macrophages directly contribute to angiogenesis in these contexts [76, 77]. This area remains to be further elucidated. Additionally, normal adult IVDs have few blood vessels and lack a direct blood supply, relying primarily on nutrient infiltration through CEPs, which makes them susceptible to degeneration. Whether neovascularization affects the blood and nutrient supply to the intervertebral disc requires further investigation.

### 3.4 Macrophage Polarization Mediates Intervertebral Disc Fibrosis

Fibrosis represents a significant pathological feature of IVDD. Typically arising from chronic inflammation, disc fibrosis results from a dynamic process of continuous damage and repair within an inflammatory microenvironment. This repair process is often irreversible, leading to pathological fibrosis in both the NP and AF [78]. In a healthy NP, the predominant cell types include large vacuolated notochordal cells, smaller chondrocyte‐like cells, collagen fibers, and aggregated proteoglycans. Degeneration of the NP involves a phenotypic shift from a notochordal to a fibroblast‐like cell state. This transition is characterized by the loss of proteoglycans and water, decreased type II collagen, and increased type I collagen, resulting in reduced hydration and elasticity, and diminished capacity to withstand mechanical stress. Similarly, while normal AF collagen fibers are well‐aligned and retain adequate proteoglycan content and water, degeneration disrupts this alignment, leading to reduced proteoglycan content, water loss, fissures, and fibrosis, which severely compromise its mechanical properties. This structural degradation facilitates pathological vascular and neural infiltration.

Macrophages play a significant role in the progression of fibrosis and are involved in the fibrotic process across various diseases, including liver, lung, and kidney fibrosis [79, 80, 81]. Recent research highlights the role of macrophages in intervertebral disc fibrosis. TGF‐β, a critical cytokine in macrophage‐mediated tissue fibrosis, exists in three isoforms (TGF‐β1, TGF‐β2, and TGF‐β3) and regulates processes such as cell proliferation, differentiation, inflammation, and fibrosis [45, 82]. Evidence suggests that TGF‐β exacerbates fibrosis in degenerating NPCs by upregulating angiopoietin‐like protein 2 [83]. Macrophages enhance the activation of TGF‐β by promoting fibrinolytic enzyme activation and by endocytosing IgG‐bound latent TGF‐β complexes, subsequently releasing active TGF‐β into the extracellular fluid. Abnormal macrophage expression of TGF‐β is linked to severe fibrosis in pulmonary and muscular tissues [84, 85]. M2 macrophages, for example, exacerbate pulmonary fibrosis through direct TGF‐β secretion, which also promotes M2 macrophage polarization [86]. Additionally, conditioned medium from M2 macrophages induces a fibrotic phenotype in NPCs, with Cemip silencing ameliorating this fibrotic phenotype [76]. These findings suggest that M2 macrophages may facilitate intervertebral disc fibrosis through TGF‐β upregulation.

Fibrinolytic enzymes and various matrix MMPs are key activators of TGF‐β. Inflammatory stimulation of M1 macrophages enhances active TGF‐β release by promoting fibrinolytic enzyme activation. MMP‐2 and MMP‐9 cleave latent TGF‐β to activate it, and M1 macrophages are known to produce high levels of MMPs in disc degeneration. Chronic inflammation mediated by M1 macrophages thus contributes to tissue fibrosis, indicating that M1 macrophages may indirectly promote intervertebral disc fibrosis. Although evidence suggests that macrophages influence disc fibrosis, direct effects have yet to be clearly demonstrated, and the underlying mechanisms remain to be fully elucidated. Further investigation is needed to clarify these mechanisms.

### 3.5 Macrophage Polarization Influences Neural Ingrowth and Sensitization

LBP is a predominant symptom of disc degeneration, largely attributed to neural ingrowth and sensitization within the degenerated disc, in addition to nerve entrapment caused by disc herniation. In healthy discs, the intact ECM prevents the inward growth of nerve fibers, which are typically confined to the outer AF and endplates [87]. However, in the degenerated state, fissures in the AF weaken these physical barriers and chemical inhibitors, such as aggregated proteoglycans, allowing sensory nerve fibers to penetrate the inner AF and NP [88, 89, 90].

Neuroinflammatory factors primarily drive inward nerve growth and sensitization. Nerve growth factor (NGF) stimulates peripheral sensory neurons to infiltrate the intervertebral disc and enhances the expression of NGF, calcitonin gene‐related peptide (CGRP), and substance P (SP) in sensory neurons, leading to neural sensitization [91]. Inflammatory factors in degenerative IVDs further stimulate NGF production in disc cells [74, 92]. M1 macrophages release NGF and pro‐inflammatory cytokines, creating a chronic inflammatory microenvironment that promotes neural ingrowth and neuroinflammation, contributing to discogenic pain [93, 94]. Additionally, TNF‐α, IL‐1β, and IL‐6 produced by M1 macrophages in degenerating discs sensitize sensory neurons to thermal and mechanical stimuli, indicating a role for M1 macrophages in promoting nerve growth and sensitization.

Conversely, M2 macrophages may mitigate nerve sensitization by modulating the inflammatory environment. IL‐10 and TGF‐β have been reported to reduce pain signaling by inhibiting the sensitization of nerve endings [95, 96, 97]. Through the secretion of IL‐10 and TGF‐β, M2 macrophages can modulate the inflammatory milieu in degenerated discs, potentially providing pain relief. Despite the supportive evidence for the pain‐relieving effects of M2 macrophages via IL‐10 and TGF‐β, direct evidence of their role in discogenic pain remains lacking and requires further investigation [98, 99].

## 4 Targeting Macrophage Polarization Therapy in IVDD

A comprehensive understanding of macrophage polarization in IVDD is essential for developing effective therapeutic strategies. Modulating macrophage polarization presents a promising approach for IVDD treatment. Various therapeutic modalities targeting macrophage polarization have been explored, including natural molecules, extracellular vesicles (EVs), bioactive materials, and gene modulation (Table 2).

**TABLE 2:** Targeting macrophage polarization therapy in IVDD.

Treatment	Classification	Experimental models (stimuli)	Effect on polarization	Signaling pathway/mechanism	Function
Nim	Natural molecule	IVDD rat mNPC(LPS, IL‐4)	M1↓ M2↑	SIRT1↑	Maintains the matrix metabolism equilibrium of NPCs
MAG	Natural molecule	hNPC	M1↓	NA	Apoptosis of NPC↓ ECM degradation↓
PRP‐derived exosomes	Evs	IVDD rat rNPC(IL‐4)	M1↓ M2↑	NF‐κB、MAPK↓ STAT6↑	Apoptosis of NPC↓
BMSC‐Exos	Evs	IVDD rats rNPC(IL‐1β)	M1↓	delivering CAHM	Apoptosis of NPC↓ ECM degradation of NPCs↓
BMSCs‐EVs	Evs	IVDD rat	M1↓	p38 MAPK↓	Apoptosis of NPC↓ ECM degradation of NPCs↓
Rapa@Gel	Bioactive material	IVDD rat	M1↓ M2↑	ROS↓	Inflammation↓
SfP4‐Mg(+) hydrogel	Bioactive material	IVDD rat rNPC	M1↓ M2↑	NA	Cell matrix synthesis↑
MnO2@TMNP	Bioactive material	IVDD rat rNPC	M1↓	ROS↓	NPC Death↓ Matrix degradation↓ Growth of Sensory Nerves↓
(OPF/SMA) hydrogel scaffold	Bioactive material	IVDD rat	M1↓ M2↑	NA	Inflammation↓ ECM synthesis↑
Silencing DNMT1	Gene modifying	IVDD rat rNPC	M1↓ M2↑	SIRT6↑	Pyroptosis of NPCs↓ NPCs proliferation↑ NPCs apoptosis↓
FCGR2A knockdown	Gene modifying	IVDD rat rNPC(LPS)	M1↓ M2↑	NF‐κB/STAT3↓	NPCs proliferation↑ Matrix degradation↓
MLT	Hormone	IVDD rat mNPC	M1↓	SIRT1↑ Notch↓	ECM anabolic↑ ECM degradation↓

Abbreviations: ECM, extracellular matrix; Evs, extracellular vesicles; IVDD, intervertebral disc degeneration; LBP, low back pain; MAG, Magnoflorine; MLT, melatonin; mNPC, mouse nucleus pulposus cells; Nim, nimbolide; NPCs, nucleus pulposus cells; rNPC, rat nucleus pulposus cells; ROS, reactive oxygen species.

### 4.1 Natural Molecule

Natural molecules, primarily derived from plants, are increasingly investigated for their therapeutic potential due to their efficacy and minimal reported side effects. Neem lactone (Nim), a citrulline analog from the neem tree ( *Azadirachta indica* ), is a highly oxidized tetracosane triterpene. In a rat model of IVDD, Nim promotes a shift toward M2 macrophage polarization by enhancing cholesterol homeostasis, thereby delaying IVDD progression by inhibiting inflammatory signaling pathways that favor M1‐like macrophage polarization [100, 101]. Magnoflorine (MAG), a plant‐derived quaternary aporphine alkaloid, has been shown to reduce LPS‐induced M1 polarization in THP‐1 cells, leading to decreased apoptosis and ECM degradation in myeloid cells [31]. Additionally, certain natural molecules, such as baicalin IV and curcumin, exhibit both macrophage polarization modulation and alleviation of disc degeneration, although direct evidence linking these effects remains limited [102, 103, 104, 105].

### 4.2 EVs

EVs are membrane‐bound particles secreted by cells into the extracellular environment, encompassing exosomes, microvesicles, and apoptotic bodies [106, 107]. Initially discovered in 1946 and officially named by E. Bonucci in 1971, EVs play a crucial role in intercellular communication and material exchange by transporting proteins, lipids, and nucleic acids [108, 109]. In recent years, EVs have emerged as a prominent focus in the treatment of IVDD.

Qian et al. demonstrated that exosomes derived from platelet‐rich plasma (PRP) inhibit M1 macrophage polarization by modulating the NF‐κB and MAPK pathways while promoting M2 macrophage polarization through the STAT6 signaling pathway. This modulation reduces the expression of inflammatory mediators and apoptotic factors, thereby mitigating the progression of IVDD [110]. Similarly, Li et al. found that exosomes from bone marrow mesenchymal stem cells (BMSC‐Exos) inhibit M1 macrophage polarization, decrease excessive apoptosis of NPCs, and prevent ECM degradation during disc degeneration [111]. Additionally, another study revealed that mesenchymal stem cell (MSC)‐derived EVs reduce M1 macrophage polarization and slow the progression of IVDD by delivering miR‐129‐5p, which targets LRG1 and subsequently activates the p38 MAPK signaling pathway [112].

Despite their therapeutic potential, several issues need addressing. These include variability between EVs derived from different cellular origins, lack of standardized dosing and duration of EV interventions, and inconclusive methods of administration. The impact of these factors on EV efficacy remains uncertain and warrants further investigation.

### 4.3 Bioactive Material

Recent studies have highlighted the potential of bioactive materials in disc repair strategies, particularly their role in modulating macrophage polarization to enhance disc cell regeneration. Therapeutic scaffolds composed of ROS (ROS)‐degradable hydrogels have been employed in damaged intervertebral discs. These hydrogels increase the proportion of M2 macrophages while decreasing M1 macrophages, thereby improving the inflammatory microenvironment and ameliorating disc degeneration [113]. Similarly, injectable hydrogels that release Mg2+ have been shown to inhibit the progression of IVDD by promoting M2 and reducing M1 macrophage polarization [114]. A nanomaterial encapsulating MnO2 nanoparticles with TrkA overexpressing macrophage membranes (TMNP) effectively scavenges intracellular ROS and prevents macrophage M1 polarization, reducing intervertebral disc inflammation and promoting matrix regeneration in a rat model of intervertebral disc injury [115]. Composite hydrogel scaffolds with IL‐4‐loaded PLGA microspheres (IL‐4‐PLGA) induce macrophages to shift from M1 to M2 phenotype at the early stage through sequential drug release in vivo, modulating the local inflammatory microenvironment and sustaining the repair of NP tissue [116].

While bioactive materials exhibit promising loading and releasing capabilities, concerns remain regarding their biocompatibility and potential immune responses to foreign materials within the human body. Additionally, data on the long‐term efficacy and safety of these bioactive materials for disc degeneration treatment are limited, necessitating further long‐term clinical studies.

### 4.4 Gene Modifying

Gene modulation represents a cutting‐edge approach in biomedical research, particularly for disease treatment, offering precision, durability, and personalization. Advances in scientific research and technology have facilitated progress in regulating macrophage polarization to address IVDD.

DNA methyltransferase 1 (DNMT1) is a key enzyme responsible for maintaining DNA methylation patterns post‐replication. Hou et al. demonstrated that inhibiting DNMT1 using lentiviral vector transfection reduced the overexpression of M2 macrophage‐specific markers CD163 and Arg‐1 in a rat model of IVDD, thereby mitigating the inflammatory microenvironment and ameliorating disc degeneration [40]. Additionally, FCGR2A, a gene encoding an immunoglobulin Fc receptor, was investigated by Luo et al., who found that FCGR2A knockdown decreased M1 macrophage polarization and NF‐κB phosphorylation while enhancing M2 polarization and STAT3 activation, resulting in improved outcomes in a rat model of IVDD [117].

Despite its potential, gene modulation faces several challenges, including high costs and technical complexity, which limit its widespread application. Ethical concerns also complicate the clinical translation of these methods. Nevertheless, the identification of specific genes as potential targets for predicting and diagnosing disc degeneration offers promising prospects for future research and therapeutic development.

## 5 Discussion

The pathogenic mechanism of IVDD involves the interplay of various pathophysiologic processes and is the fundamental pathologic underpinning of chronic LBP. According to recent research, macrophage polarization is essential for controlling the processes of fibrosis, pathologic angiogenesis, and NPC senescence. These processes work together to create an unbalanced disc microenvironment through a complex web of cellular molecules. The dynamic relationship between macrophage polarization and NPC senescence, angiogenesis, and fibrosis is used in this retrospective literature review to methodically examine the crucial role that macrophage polarization plays in the development of IVDD.

An early hallmark event of IVDD is NPCs senescence, which is characterized by an imbalance in the synthesis and degradation of ECM and the activation of senescence‐associated secretory phenotypes (SASPs) [13]. It has been established that M1 macrophages trigger the p38 MAPK/p16INK4a signaling axis by releasing pro‐inflammatory substances like TNF‐α and IL‐1β, which markedly increase β‐galactosidase activity and p53/p21 expression in NPCs, resulting in telomere shortening and mitochondrial dysfunction [112]. NPCs in the M1‐polarized microenvironment displayed typical aging morphological characteristics, such as nuclear membrane folds and lysosomal proliferation, according to transmission electron microscopy observations [57]. On the other hand, M2‐type macrophages efficiently scavenged ROS and preserved mitochondrial homeostasis by upregulating the expression of SIRT1 deacetylase and activating the PI3K/AKT/mTOR pathway in NPCs by secreting IL‐4/IL‐13 [118]. According to single‐cell sequencing data, M2 conditioned media increased COL2A1 and Aggrecan production by 3.1 times while decreasing the expression of genes linked to senescence by 62% [46, 119].

The development of pain is directly linked to abnormal vascular invasion of the intervertebral disc, and macrophage polarization is precisely regulated in this process both spatiotemporally and geographically. Through NF‐κB‐dependent pathways, M1 macrophages increase the production of VEGF, PDGF, and MMP‐9, which facilitates capillary bud development and vascular endothelial cell migration [120]. Notably, the Semaphorin 3A/Neuropilin‐1 axis may work in concert with nerve growth factor (NGF) released by M1 macrophages to cause aberrant perivascular nerve fiber development, which would lead to the phenomenon known as “vascular‐neural coupled invasion [121]”. “By secreting TIMP‐1 and soluble VEGFR1, on the other hand, M2 macrophages competitively inhibited angiogenic signaling.” They did this by down‐regulating the expression of CEMIP (hyaluronidase‐regulated gene), which effectively maintained the anti‐angiogenic barrier of medullary tissue and reduced hyaluronic acid degradation by 58% [122]. Intervertebral disc vascular density and M1/M2 ratio were significantly positively correlated in patients with advanced IVDD, according to immunohistochemistry of clinical specimens [30].

The terminal phase of IVDD is characterized by ECM fibrotic remodeling, which is influenced by macrophage subtypes through differential regulation of the TGF‐β signaling pathway [85]. Through the ROS/STAT3 pathway, type M1 triggers the TGF‐β1/Smad2/3 cascade, which encourages NPCs to transdifferentiate into myofibroblasts and results in aberrant type I/III collagen deposition (a 4.3‐fold increase). M1‐conditioned media increased collagen contraction to 78% ± 5%, according to three‐dimensional collagen contraction studies [123]. M2 type, on the other hand, inhibited TGF‐β signaling overactivation, increased collagenase activity by miR‐29b overexpression, and activated Smad7 inhibitory factor through IL‐10 secretion. The M2c subpopulation notably expresses the ED‐A isoform of fibronectin, which may be implicated in the dynamic balance of fibrotic microenvironmental remodeling, according to single‐cell transcriptome study.

Natural polyphenolic compounds (like resveratrol) reprogramme M2 polarization through the Nrf2/ARE pathway [124]; engineered exosomes that deliver miR‐223‐3p to inhibit NLRP3 inflammatory vesicles; and bionic scaffolding materials that modulate the local mechanistic microenvironment inducing M2 transformation are some of the current therapeutic strategies that target macrophage polarization [125]. Three main obstacles stand in the way of clinical translation, though: first, M2 isoforms may inhibit inflammation while promoting angiogenesis through the IGF‐1 pathway, necessitating the development of spatiotemporal‐specific modulation techniques; second, polarization phenotypes dynamically change at different stages of degeneration, making it challenging to sustain the long‐term effectiveness of a single intervention; and third, the metabolic features of the avascular microenvironment of human intervertebral discs cannot be fully replicated by the animal models currently in use. To examine the exact molecular mapping of the interaction between macrophages and NPCs, future studies must combine organoid culture techniques with single‐cell spatial transcriptomics. To create a new avenue for the accurate treatment of IVDD, intelligent responsive biomaterials should be created that can dynamically regulate the polarization state in response to variations in the pH of the microenvironment and mechanical loads.

## 6 Conclusions

The dynamic control of macrophage polarization is intimately linked to the fundamental pathogenic mechanism of IVDD. While M2‐type macrophages activate the PI3K/AKT/mTOR pathway through IL‐4/IL‐13 to maintain NPC homeostasis, M1‐type macrophages activate the p38 MAPK pathway through the release of TNF‐α/IL‐1β to induce myeloid cell (NPC) senescence, which is characterized by elevated β‐galactosidase activity and metabolic imbalance of the extracellular matrix. By blocking CEMIP, M2 type preserves the antivascular barrier during angiogenesis, whereas M1 type encourages vascular‐neural connection invasion through VEGF/MMP‐9. M1 type increases TGF‐β/Smad signaling through ROS/STAT3 to induce aberrant collagen deposition in the fibrotic process, while M2 type prevents fibrosis through IL‐10/miR‐29b. The functional complexity of M2 subtypes, polarization dynamics, and clinical translational obstacles confront current therapeutic approaches that target polarized phenotypes (e.g., exosomal delivery of miRNAs, bionanomaterials). To achieve spatiotemporal precision intervention and offer a fresh approach to IVDD treatment, single‐cell histology and smart material technologies must be combined in the future.

## Author Contributions

**Kaiyuan Zheng:** data analysis, writing – original draft, funding acquisition. **Siyu Wang:** data analysis. **Meng Deng:** methodology, supervision. **Yaomin Luo:** image analysis. **Wen Li:** image analysis. **Lianlin Zeng:** writing – review and editing. **Yinxu Wang:** conceptualization, methodology, supervision. All authors participated in this article.

## Ethics Statement

The authors have nothing to report.

## Consent

The authors have nothing to report.

## Conflicts of Interest

The authors declare no conflicts of interest.

## Data Availability

All data are in the manuscript and/or Supporting Information files.
